# Rabies is still a fatal but neglected disease: a case report

**DOI:** 10.1186/s13256-021-03164-y

**Published:** 2021-12-01

**Authors:** Y. A. Amoako, P. El-Duah, A. A. Sylverken, M. Owusu, R. Yeboah, R. Gorman, T. Adade, J. Bonney, W. Tasiame, K. Nyarko-Jectey, T. Binger, V. M. Corman, C. Drosten, R. O. Phillips

**Affiliations:** 1 Kumasi Centre for Collaborative Research, Kwame Nkrumah University of Science and Technology, Kumasi, Ghana; 2 Department of Medicine, School of Medicine and Dentistry, Kwame Nkrumah University of Science and Technology, Kumasi, Ghana; 3 Institute of Virology, Charite Universitatsmedizin Berlin, Berlin, Germany; 4 Department of Theoretical and Applied Biology, Kwame Nkrumah University of Science and Technology, Kumasi, Ghana; 5 Department of Medical Laboratory Technology, College of Health Sciences, Kwame Nkrumah University of Science and Technology, Kumasi, Ghana; 6 Obuasi Municipal Health Directorate, Obuasi, Ghana

**Keywords:** Rabies, Incubation period, Diagnostic testing, Ghana

## Abstract

**Background:**

Rabies, caused by a lyssavirus, is a viral zoonosis that affects people in many parts of the world, especially those in low income countries. Contact with domestic animals, especially dogs, is the main source of human infections. Humans may present with the disease only after a long period of exposure. Nearly half of rabies cases occur in children <15 years old. We report on a fatal case of rabies in a Ghanaian school child 5 years after the exposure incident, and the vital role of molecular tools in the confirmation of the diagnosis.

**Case presentation:**

The patient, an 11-year-old junior high school Ghanaian student from the Obuasi Municipality in Ghana, presented with aggressive behavior, which rapidly progressed to confusion and loss of consciousness within a day of onset. Her parents reported that the patient had experienced a bite from a stray dog on her right leg 5 years prior to presentation, for which no antirabies prophylaxis was given. The patient died within minutes of arrival in hospital (within 24 hours of symptom onset). Real-time polymerase chain reaction testing of cerebrospinal fluid obtained after her death confirmed the diagnosis of rabies. Subsequent phylogenetic analysis showed the virus to belong to the Africa 2 lineage of rabies viruses, which is one of the predominant circulating lineages in Ghana.

**Conclusion:**

The incubation period of rabies is highly variable so patients may only present with symptoms long after the exposure incident. Appropriate molecular testing tools, when available as part of rabies control programmes, are vital in confirming cases of rabies.

## Background

Rabies, a viral zoonosis caused by a lyssavirus, is a vaccine-preventable, neglected tropical disease (NTD) that occurs in more than 150 countries and territories [1]. In the USA, the majority of rabies cases reported to the Centers for Disease Control and Prevention (CDC) each year occur in wild animals such as bats, raccoons, skunks, and foxes [2]. However, rabies can affect any mammal, including humans. Dogs are the main source of human rabies deaths, contributing up to 99% of all rabies transmissions to humans. The rabies virus infects the central nervous system of mammals, ultimately causing disease in the brain and death. Infection causes tens of thousands of deaths every year, mainly in Asia and Africa. Approximately 40% of people bitten by suspect rabid animals are children under 15 years of age. Rabies elimination is feasible through vaccination of dogs and prevention of dog bites [1]. In Ghana, rabies remains an important public health threat, with case fatality rate of 100% [3–6].

We report on a case of rabies in an 11-year-old Ghanaian student and discuss the essential role of accurate diagnosis in rabies control.

## Case presentation

The patient was an 11-year-old junior high school Ghanaian student from the Obuasi Municipality in Ghana, with no known previous illnesses. She was the third child from a family of five children. She presented with a day’s history of aggressive behavior, which rapidly progressed to confusion and loss of consciousness. Her parents reported that the patient had experienced a bite from a stray dog on her right leg 5 years prior to presentation. Additionally, her parents described episodes of hydrophobia within the preceding year; however, they did not make much of it as they considered it to be mild and due to the ‘playful nature of children’. Other prodromal symptoms such as fever, general malaise, sore throat, anorexia, and muscle weakness were absent. There was no history of dysphagia. The patient was brought to the hospital within a day of onset of her symptoms and died from cardiorespiratory failure within minutes of arrival in hospital (within 24 hours of symptom onset). Initial assessment revealed an ill-looking, unconscious patient, who was afebrile with a respiratory rate of 14 cycles per minute. The neck was supple and Kernig’s sign was negative. The pupils were dilated and sluggishly reactive to light. Real-time polymerase chain reaction (RT-PCR) testing of cerebrospinal fluid obtained after her death confirmed the diagnosis of rabies. The corpse was handled and buried/disposed in accordance with standard local protocols in Ghana [7]. The contacts of the patient were subsequently counseled and offered rabies vaccination. All the contacts of the patient were free of rabies symptoms after 12 months of follow-up.

### Virus characteristics

Given the reported long incubation period of the case, sequencing and phylogenetic analysis of the detected virus was performed to assess the possibility of another origin of the virus other than the reported dog bite, such as from bats, which may not have been recognized. Viral Ribonucleic acid (RNA) was extracted from the patient’s cerebrospinal fluid (CSF) using the Qiagen Viral RNA mini spin kit (Qiagen, Hilden, Germany), according to the manufacturer’s instructions. Presence of rabies RNA was confirmed by RT-PCR testing using a Lyssa-Virus RT-PCR kit (Tib Molbiol, Berlin, Germany) and a LightCycler Multiplex RNA Virus Master (Roche, Penzberg, Germany). We applied a high-throughput sequencing (HTS) approach for whole genome sequencing using the KAPA RNA Hyper Prep Kit (Roche Molecular Diagnostics, Basel, Switzerland) for library preparation and the 150-cycle NextSeq reagent v3 cartridge (Illumina, San Diego, California, US), according to manufacturer’s instructions.

Bidirectional reads from the HTS run were assembled against a reference rabies sequence from GenBank and annotated using Geneious prime 2019 (https://www.geneious.com). Phylogenetic analysis was done by maximum likelihood reconstruction using the PHYML [8] plugin in Geneious prime with 500 bootstrap replicates.

**Fig. 1.**
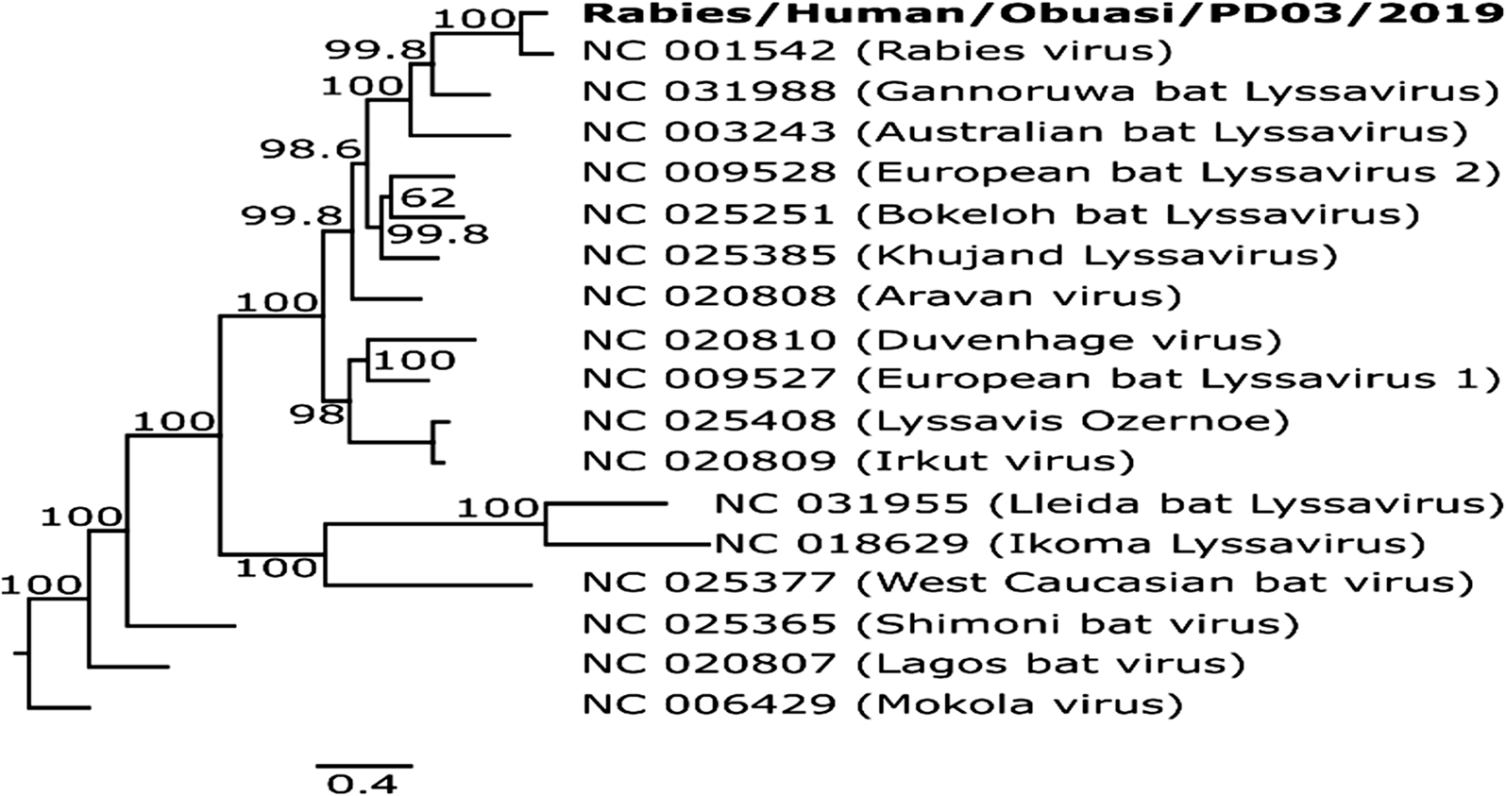
Phylogenetic tree comparing Lyssavirus genotypes. Tree was generated using maximum likelihood reconstruction by the general time reversible model with a gamma distribution and proportion of invariable sites (GTR+I+G). The tree is based on whole genome sequences and was rooted with a Mokola virus (Genotype 3). Tips were labeled with accession numbers and virus names in brackets. The sequence obtained in this study is shown by bold type font

**Fig. 2.**
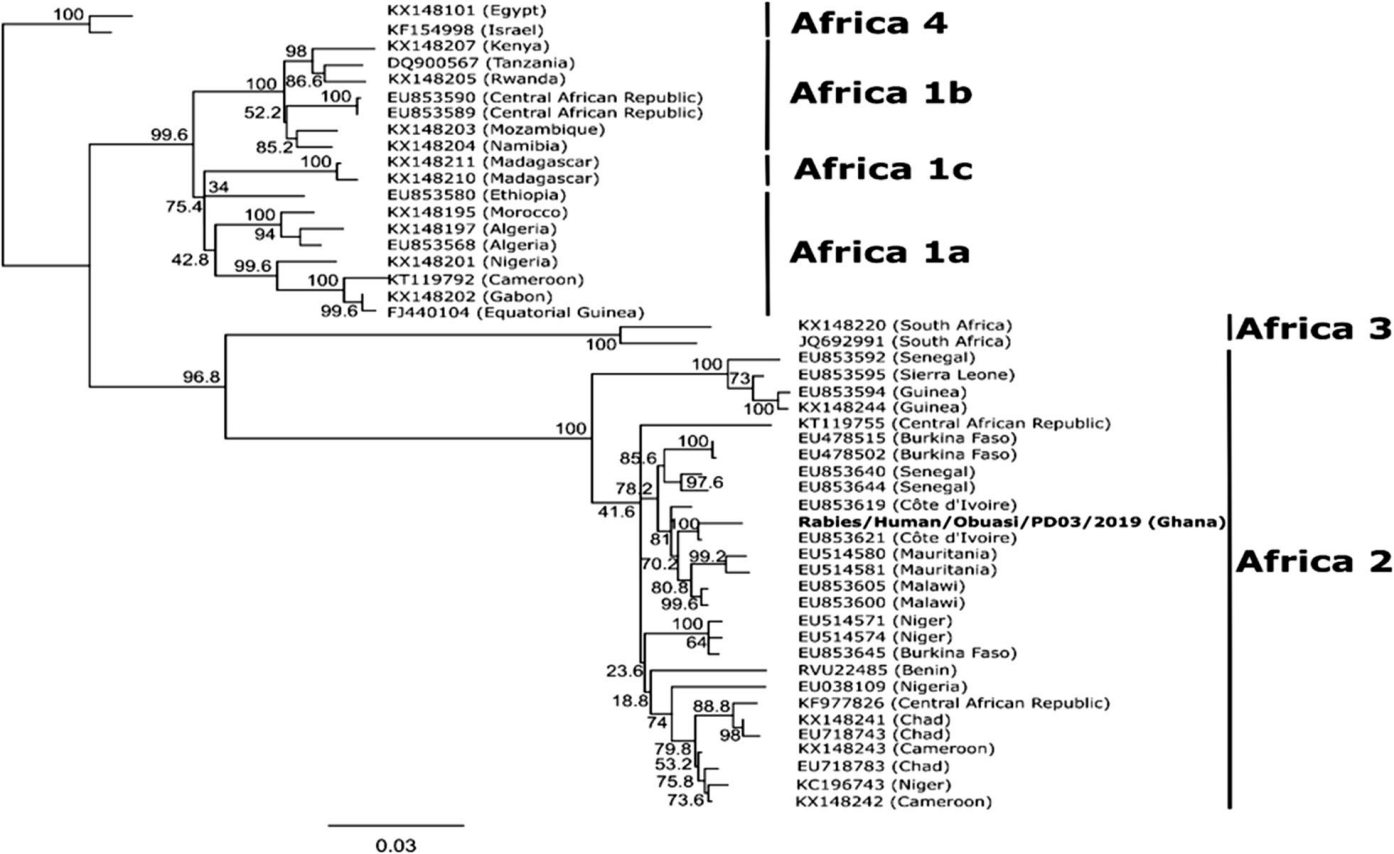
Phylogenetic tree comparing rabies viruses from Africa. Tree was generated using maximum likelihood reconstruction by a transition model with a gamma distribution and proportion of invariable sites (TIM1+G+I). The tree is based on complete nucleoprotein sequences and rooted with the Africa 4 lineage branch. Tips are labeled with accession numbers and country of origin in brackets. The sequence obtained in this study is shown by a bold type font

The sequence obtained was found to be most closely related to a Rabies virus (Accession number: NC_001542), sharing an 85.4% pairwise sequence identity and forming a monophyletic pairing with this virus when compared with other reference Lyssaviruses from GenBank (Fig. 1). Comparison of the full nucleoprotein coding region to those of a representative subset of African rabies viruses of various lineages [9] showed the virus to belong to the Africa 2 lineage of rabies viruses, which is one of the predominant circulating lineages in Ghana [10] (Fig. 2). The full genome obtained in this study was submitted to GenBank and assigned accession number MT107888.

### Ethical considerations

Ethical approval for this study was obtained from the Scientific and Ethical review Committee of the School of Medical Sciences, Kwame Nkrumah University of Science and Technology (KNUST) (CHPRE/AP/462/19). Written informed consent was also obtained from parent of the patient for publication of this case report.

## Discussion

Rabies is a neglected tropical disease of poor and vulnerable populations, with deaths due to rabies often not reported. Rabies is nearly always fatal once symptoms appear. Although 100% preventable, over 59,000 people, mostly in under-served areas, in over 150 countries, die of rabies every year as human vaccines and immunoglobulin are not readily available or accessible [1, 5]. Operationally in Ghana [7, 11], all cases of dog bites are considered suspected cases of rabies, and a confirmed case is defined as a suspected case with clinical and or laboratory confirmation. The clinical confirmation of rabies is based on a history of dog bite that is followed by classical symptoms such as anxiety, agitation, paralysis, excessive salivation, and hydrophobia. The patient presented in this report met the case definition and tested positive by PCR and subsequent phylogenetic analysis as described.

The incubation period for rabies is typically 2–3 months but may vary from 1 week to 1 year. This period may vary based on the location of the exposure site (how far away it is from the brain), the type of rabies virus, viral load, and any existing immunity [1]. Rabies causes an acute progressive viral encephalomyelitis. The first symptoms of rabies may be very similar to those of flu including general weakness or discomfort, fever, or headache. The disease may present as furious or paralytic rabies. Furious rabies presents with signs of hyperactivity, excitable behavior, hydrophobia, and sometimes aerophobia. Death occurs after a few days due to cardio-respiratory arrest. Paralytic rabies accounts for about 20% of the total number of human cases and runs a less dramatic and usually longer course than the furious form. Muscles gradually become paralyzed, starting at the site of the bite or scratch. A coma slowly develops, and eventually death occurs. The paralytic form of rabies is often misdiagnosed, contributing to the under-reporting of the disease.

In a previous study from Ghana [3], the time between exposure and the onset of symptoms ranged between 3 weeks and 4 months, with 52.4% of cases reporting the onset of symptoms approximately 2 months after exposure. There was a history of a dog bite about 5 years prior to the onset of symptom in the patient presented in this report; this represents a rather long incubation period and was possibly influenced by recall bias from the parents who gave the clinical history. A more reasonable scenario will be that the patient had some further exposure to the rabies virus. The lineage of the detected virus implicates a common circulating rabies virus, most likely from dogs, and suggests she could have been innocuously exposed to the saliva or been scratched by an infected stray dog in the immediate period preceding her demise; but we have no way of confirming this. This assessment is in line with that of another study in Bangladesh that also found three patients with reported incubation period in excess of 1000 days, which was attributed to recall bias and likely recurrent exposures following the first bite incident [12]. A further possibility is that the virus was replicating slowly with the establishment of a latent infection following the initial exposure 5 years earlier, with subsequent reactivation of the neurotropic virus infection in later years. Although rare, long incubation period for rabies have been reported. Shankar and colleagues [13] reported a case of rabies encephalitis with a possible 25 year incubation period and suggested that reactivation of a latent infection may have played a role in the pathogenesis of the disease. In that study, the diagnosis of rabies was established by histopathology. An incubation period longer than > 6.5 years was reported in a 10-year-old girl of Vietnamese origin in whom rabies developed after she had lived continuously in Australia for almost 5 years [14]. Viral molecular epidemiological tools as used in our study provide insight into the migratory pattern of the virus-carrying animal and human vectors, but not the mechanism of viral latency [14, 15].

In Ghana, rabies is endemic and cases of human rabies are under reported, as in other developing countries [1, 6]. Twenty-one cases of rabies were seen at a tertiary facility in Ghana over a 25 month period, with more than half of cases aged >18 years [3]. Among that population, hydrophobia and agitation were the most common symptoms, and the case fatality rate was 100% with about 60% of cases dying within 24 hours of admission. The longest duration of stay recorded in that study was 5 days. Our patient had similar symptoms and died within 24 hours of hospitalization, in keeping with the aggressive course of the illness.

The veterinary services in Ghana are often limited in the diagnosis of rabies, as Sellers’ stain and fluorescent antibody test, commonly used techniques in diagnosing rabies, are mostly unavailable. The clinical diagnosis of human rabies is partly based on a positive rabies test result of the offending animal from the veterinary services. Preventive and control measures in Ghana to reduce the incidence of human rabies have been targeted at improving the vaccination of dogs against rabies, stray dog removal, and providing pre-/ post-exposure vaccinations of humans; however, these measures have been irregular and not sustained [6, 16].

The differential diagnosis for this case includes other etiologies of central nervous infections (such as bacterial, fungal, and other viruses and abscess) and intracranial tumors. Although these other etiologies were not sought for, the classic clinical presentation, together with the PCR and phylogenetic characterization of the rabies virus in the patient’s CSF make these other differential etiologies less likely.

Laboratory diagnosis of rabies infection in humans is difficult after exposure to the virus before the onset of clinical symptoms. Clinical diagnosis of rabies is often made when rabies-specific signs, such as hydrophobia or aerophobia, are present. Human rabies can be confirmed during clinical disease stage and postmortem by detecting viral antigens, whole virus, or nucleic acids in infected tissues (brain, skin, urine, or saliva) using various diagnostic techniques[1]. Accurate diagnosis of rabies in exposed persons will enable the institution of appropriate care. In the incident case, rabies confirmation by PCR testing of cerebrospinal fluid only occurred after death of the patient. In under-served populations where the threat of rabies is highest, diagnostic facilities are largely absent, thus hampering early and accurate diagnosis. Indeed, in most cases, the diagnosis is only presumptively made on clinical grounds [3, 17]. Over the past several years, the Kumasi Centre for Collaborative Research in Tropical Medicine (KCCR) has provided support services in the area of rabies diagnosis to the Ashanti Regional Directorate of Health Services (RDHS) in Ghana. The KCCR performs PCR testing on samples received from the RDHS. Such collaborations are essential in facilitating diagnosis and guiding treatment decisions and rabies control efforts within the country.

The rabies strain reported in this case is presumably from a domestic animal (dog) contact. This is in keeping with previous accounts from Ghana [3, 6, 16] and globally [1] that implicate domestic dogs as being responsible for most rabies virus transmission to humans. A recent study in the Ashanti region of Ghana showed a high dog to household ratio, and that 80.3% of the dogs were not restricted and 49.9% were allowed to enter neighbors' households [18]. In that same study, dog rabies vaccination coverage was low, ranging from 28.1% to 64.9%. This calls for improved efforts to target the vaccination of all dogs in Ghana to prevent spread from stray animals.

While the knowledge of rabies transmission is high in Ghana, about 65% of people studied in a peri-urban setting believed in traditional ways of treatment such as concoctions, herbs, and consumption of the offending dogs’ organs [18]. This practice has the tendency to delay access to care for people exposed to rabies and contribute to rabies mortality within the Ghanaian population. Even after exposure, the tragic loss of lives from rabies is preventable, since effective post-exposure prophylaxis (PEP) is available in the form of wound care, rabies immunoglobulin, and rabies vaccine. Rabies immunoglobulin in addition to rabies vaccine is indicated for category III exposures, which include bites or scratches that penetrate the skin (as occurred in this case), licking of mucous membranes or broken skin, and direct contact with bats. Laryea and colleagues [3] reported that most patients do not seek care after exposure to rabies. Even for the third of people who seek care post exposure, they do not get access to the recommended PEP. In an Ethiopian study, 77% of suspected rabid dog bite victims visited a health center, and 57% received sufficient doses of PEP. The likelihood of seeking medical services following rabies exposure was higher for high-income earners, people bitten by dogs of unknown ownership, where the bite was severe especially on the leg, and where the victim lived close to the nearest health center [19]. Increasing access to health facilities delivering post-exposure services can lead to improved health-seeking behavior in patients following rabies exposure and reduce the mortality associated with rabies.

Rabies control efforts requires concerted collaboration between agencies in multiple sectors. In Ghana, a parallel and uncoordinated system of rabies surveillance is maintained by the health and veterinary services, with gross disparities in the number of reported events and an overall impression of under-reporting [11]. Tackling the scourge of a zoonosis such as rabies using the ‘One Health Approach’ requires a collaborative and multi-disciplinary effort that cuts across the boundaries of animal, human, and environmental health to undertake risk assessments, and to develop plans for response and control [20, 21]. The WHO, the World Organisation for Animal Health (OIE), the Food and Agriculture Organization of the United Nations (FAO), and the Global Alliance for Rabies Control (GARC) have established a global multi-sectoral “United Against Rabies” collaboration to provide a common strategy to achieve "Zero human rabies deaths by 2030" [22]. In Ghana, such multi-sectoral collaboration will involve the Ministry of Health and public health authorities, veterinary services, Ministry of Local Government and Rural Development, and Municipal and district assemblies among others, as important stakeholders.

The median age of rabies victims in a previous study [11] from Ghana was 9 years (range 3–72 years) and the patient presented in this report was aged 11 years. Since the at-risk population includes a large proportion of children of school going age, an important control measure might be increasing awareness of rabies in this age group. In a Malawian study, knowledge of rabies and how to be safe around dogs was greater among school children who had received a school lesson on rabies compared with those who had not received the lesson, but had been exposed to a rabies vaccination campaign in their community (both p < 0.001), indicating that the lesson itself was critical in improving knowledge [23]. The primary school education curriculum should include basic content to educate young children on the dangers of an animal bite and encourage them to seek help. Education in rural and urban communities targeting community leaders, chiefs, farmers, pet owners, and schools on rabies prevention will create awareness among the public and aid rabies control efforts.

## Conclusion

The incubation period of rabies is highly variable, so patients may only present with symptoms long after the incident exposure. Rabies should be considered in the differential diagnosis of patients who present with encephalopathy. Appropriate molecular testing tools are vital in confirming and documenting cases of rabies in people who meet the case definition. There is a need to increase knowledge and awareness of rabies and provide appropriate post-exposure prophylaxis to reduce the incidence of human rabies and the associated fatalities.

## Data Availability

The datasets obtained and analysed during the current study are available from the corresponding author on reasonable request. The full genome obtained in this study was submitted to GenBank and assigned accession number MT107888.

## Abbreviations

NTD: Neglected tropical disease
CDC: Centers for Disease Control
PCR: Polymerase chain reaction
RT-PCR: Real-time polymerase chain reaction
RNA: Ribonucleic acid
HTS: High-throughput sequencing
CSF: Cerebrospinal fluid
PEP: Post-exposure prophylaxis
OIE: World Organisation for Animal Health
FAO: Food and Agriculture Organization
GARC: Global Alliance for Rabies Control
